# Mechanical ventilation preserves diaphragm mitochondrial function in a rat sepsis model

**DOI:** 10.1186/s40635-021-00384-w

**Published:** 2021-04-07

**Authors:** P. Eyenga, D. Roussel, B. Rey, P. Ndille, L. Teulier, F. Eyenga, C. Romestaing, J. Morel, V. Gueguen-Chaignon, S-S. Sheu

**Affiliations:** 1 Center for Translational Medicine, Department of Medicine, Thomas Jefferson University, Philadelphia, PA 19107 USA; 2 Laboratoire d’Ecologie des Hydrosystèmes Naturels et Anthropisés, UMR 5023, Université de Lyon, Université Lyon1, CNRS, 69622 Villeurbanne, France; 3 Laboratoire de Biométrie et Biologie Evolutive, UMR 5558, Université de Lyon, Université Lyon1, CNRS, 69622 Villeurbanne, France; 4 Protein Science Facility, ENS de Lyon, Inserm, US8, SFR Biosciences UMS 3444 - CNRS Université Claude Bernard Lyon 1, 69007 Lyon, France; 5 Université Claude Bernard Lyon 1, 69008 Lyon, France; 6 Département de Chirurgie, Centre Hospitalier D’Ebomé, Kribi, Cameroun; 7 Service de réanimation chirurgicale, CHU de Saint Etienne, 42000 Saint Etienne, France

**Keywords:** Sepsis, Diaphragm, Mechanical ventilation, Mitochondria, Cytochrome c oxidase, Oxidative stress, Interleukin-1β

## Abstract

**Background:**

To describe the effect of mechanical ventilation on diaphragm mitochondrial oxygen consumption, ATP production, reactive oxygen species (ROS) generation, and cytochrome c oxidase activity and content, and their relationship to diaphragm strength in an experimental model of sepsis.

**Methods:**

A cecal ligation and puncture (CLP) protocol was performed in 12 rats while 12 controls underwent sham operation. Half of the rats in each group were paralyzed and mechanically ventilated. We performed blood gas analysis and lactic acid assays 6 h after surgery. Afterwards, we measured diaphragm strength and mitochondrial oxygen consumption, ATP and ROS generation, and cytochrome c oxidase activity. We also measured malondialdehyde (MDA) content as an index of lipid peroxidation, and mRNA expression of the proinflammatory interleukin-1β (IL-1β) in diaphragms.

**Results:**

CLP rats showed severe hypotension, metabolic acidosis, and upregulation of diaphragm IL-1β mRNA expression. Compared to sham controls, spontaneously breathing CLP rats showed lower diaphragm force and increased susceptibility to fatigue, along with depressed mitochondrial oxygen consumption and ATP production and cytochrome c oxidase activity. These rats also showed increased mitochondrial ROS generation and MDA content. Mechanical ventilation markedly restored mitochondrial oxygen consumption and ATP production in CLP rats; lowered mitochondrial ROS production by the complex 3; and preserved cytochrome c oxidase activity.

**Conclusion:**

In an experimental model of sepsis, early initiation of mechanical ventilation restores diaphragm mitochondrial function.

## Background

During sepsis or septic shock, the lung is one of the first organs to undergo dysfunction, and acute lung injury or acute respiratory distress syndrome related to sepsis contributes greatly to poor outcomes [1]. Sepsis-induced diaphragmatic dysfunction could also contribute to respiratory failure in this setting. Indeed, septic laboratory animals have been shown to die of respiratory failure related not to pulmonary disease per se, but rather to the failure of the diaphragm to ventilate the lungs [2]. Several mechanisms could explain contractile dysfunction of diaphragm muscle during sepsis [3]. Mitochondrial dysfunction and oxidative stress that lead to diaphragm protein degradation and muscle atrophy have been proposed [4]. In the septic diaphragm, spontaneous contractions that persist to sustain respiration potentially aggravate the injuries induced by oxidative and nitrosative stress [5]. The combined effect of sepsis and repeated respiratory muscle contractions has led researchers to question whether early initiation of mechanical ventilation (MV) could minimize the muscle injury and respiratory failure and improve patient outcomes [6, 7]. However, the effect of a short-term MV on diaphragm function has produced conflicting results. A previous experimental study has shown transient beneficial effect of MV on diaphragm function [8], which may be absent in the short term [9] and prolonged MV [10] in the endotoxemia model of sepsis. However, it is still unclear if ROS generated by the mitochondrial electron transport chain alteration are involved in diaphragm failure related to sepsis and MV. A recent clinical study has shown that mitochondrial function is unaffected by prolonged MV [11], while an animal model of sepsis and short-term MV has shown the opposite result [9]. If mitochondria dysfunction was not involved in diaphragm weakness, the ROS generated during sepsis [12] and MV [13] would not be decreased by anti-oxidant infusion [12, 14].

Therefore, we hypothesized that the potential beneficial effect of MV on diaphragmatic function may lie at the mitochondrial level. The present study aimed to investigate the effects of MV on diaphragm mitochondrial function during sepsis in rats subjected to cecal ligation and puncture (CLP). In particular, we investigated the oxygen consumption and adenosine triphosphate (ATP) production capacity of mitochondria isolated from diaphragm. We also measured mitochondrial ROS generation and malondialdehyde (MDA) content as a proxy for oxidative damage to lipids and investigated their relationship to diaphragm force. Finally, to get more insight on the mechanisms involved in mitochondrial dysfunction during sepsis and the preservative effects of MV, we measured the activity and content of the cytochrome c oxidase, an enzyme deeply involved in mitochondrial respiration and ROS generation [15–18].

## Methods

In this study, we used 24 male Wistar rats (280–350 g) obtained from IFFA-CREDO (L’Arbresle, France). The rats were maintained under 12:12 h artificial light–dark cycles (23 °C ± 1 °C room temperature, 30–60% relative humidity) and received a standard rat diet and water ad libitum. They were allowed to adapt to laboratory conditions for at least 1 week before the experiments started. The experimental protocol was approved by an authorized animal care laboratory of the French Health Authority and University Research Committee.

### Animal procedures

The experiments were carried out in a rat model of sepsis, as previously described [19–21]. In both sham-operated (*n* = 12) and cecal ligation and puncture (CLP) (*n* = 12) groups, anesthesia was induced by intraperitoneal injection of sodium pentobarbital (56 mg.kg^−1^) and fentanyl (20 µg.kg^−1^) and further maintained by continuous infusion of sodium pentobarbital (56 mg.kg^−1^.h^−1^) and fentanyl (20 µg.kg^−1^.h^−1^) administered in saline 0.9%. The total saline volume infused was 10 ml.kg^−1^.h^−1^ via a catheter (polyethylene 50 [PE-50]) inserted in the left jugular vein.

Half of the CLP and control rats were then chemically paralyzed and underwent MV (*n* = 6 per group). To limit excessive muscle activity causing a drop in lung compliance and increased airway pressure [22], MV rats were paralyzed using a synthetic non-depolarizing neuromuscular blocking agent (atracurium besilate, 0.3 mg.kg^−1^) administered every 2 h via a jugular catheter. The trachea was then cannulated, and the rats were intubated and ventilated with a ventilator (7025 Rodent Ventilator; Ugo Basile, Comerio, Italy) equipped with standard sterile tubing and filters. The ventilator was placed in the assist-control mode, with a trigger threshold of 0.25 cm H_2_O, and the initial settings were as follows: respiratory frequency, 90 respiratory cycles per minute; tidal volume, 6 mg.kg^−1^; fractional inspired oxygen, 0.50; and positive end-expiratory pressure, 1.5 cm H_2_O. Ventilation was adjusted to ensure an arterial PaCO_2_ of 37–42 mm Hg. The absence of respiratory muscle effort was confirmed by the absence of triggering, as well as by the stable and reproducible shape of the tracheal pressure waveform throughout the experiment.

Systemic blood pressure was monitored using a pressure transducer, which was connected to a PE-50 tubing catheter inserted in the right carotid artery. The mean arterial pressure was continuously recorded using a multichannel recording system (Biopac Systems, Santa Barbara, CA, USA). Body temperature was monitored via an intrarectal thermometer and maintained at 37.5 °C by the heat pad under the animal.

The protocol of CLP was performed to induce sepsis as described previously [19–21]. After a stabilization period of 30 min, under an aseptic condition, a 3-cm-long abdominal midline incision was made to expose the cecum at the adjoining intestine in 12 rats of the CLP groups. The cecum was tightly ligated just below the ileocecal junction without the obstruction of the bowel, three 18-gauge needle punctures on the antimesenteric border were performed, and gentle pressure was applied to the cecum until a small amount of feces exuded. The whole bowel was returned into the abdominal cavity, and the abdomen was closed with 4:0 silk. The sham-operated control rats (*n* = 12) underwent a 3-cm-long midline incision, a nontraumatic manipulation of the bowel was made, and the wound was closed with 4:0 silk. Animals were then randomly assigned to one of the following four groups, with six animals in each: (1) a spontaneously breathing control group (control); (2) a spontaneously breathing sepsis group (sepsis); (3) a mechanically ventilated control group (MV-control), and (4) a mechanically ventilated sepsis group (MV-sepsis).

Six hours after the surgical procedure, arterial blood was sampled to measure PaO_2_, PaCO_2_, pH, and HCO_3_ values. Additional blood samples were obtained by cardiac puncture for all other measurements. Then, the entire diaphragm was collected and divided in three. A first part was used to isolate the mitochondria (see mitochondria extraction protocol below). A second part from a middle part of lateral costal region of the diaphragm with fibers attached to a portion of ribs and distally to central tendon was immediately stored in Krebs solution (137 mM NaCl, 4 mM KCl, 1 mM MgCl_2_, 1 mM KH_2_PO_4_, 12 mM NaHCO_3_, 2 mM CaCl_2_, and 6.5 mM glucose) bubble with a gas mixture of 95% O_2_ and 5% CO_2_. The remaining diaphragm was stored at  −  80 °C for further analysis.

### Measurements of arterial blood gases and plasma lactic acid

Arterial blood gases were analyzed using a radiometer (ABL5; Radiometer, Copenhagen, Denmark). Plasma lactic acid was measured by an enzymatic reaction (Hitachi 712-Roche Diagnostics, Meylan, France).

### In vitro diaphragm contractile assessment

Diaphragm muscle stretch was studied with a device and protocol described and provided by Allard B [23] with several modifications. A bundle from middle part of lateral costal region of the diaphragm was dissected and the tendinous end was left intact while the other end was cut free of the rib and ligated with a fine copper wire for use in mounting preparation. The preparations were perfused with continually flowing Kreb’s solution which prevented alterations of salinity and allowed rapid elimination of toxic metabolites released from muscles. The bundles (19–22 mg) were situated between flanking platinum electrodes driven by a biphasic stimulator (6002 stimulator Harvard Apparatus, Holliston, MA, USA) connected in series with a power amplifier.

Maximum tetanic force was determined by stimulating the muscle bundles at various frequencies (30, 70, 100 Hz), delivered in 1-s duration train at optimum length for force production. Force signal was measured with a 5734 RCA transductor and monitored with 565 storage oscilloscope (Tektronix Inc., Beaverton, OR) and displayed simultaneously on a BD 111. chart recorder (Kipp & Zonen, Delft., The Netherlands). Isometric force was determined at 70 Hz across the range of stimulation frequencies because a previous study [24, 25] showed that this stimulation frequency is optimal for generating peak tetanic forces. Isometric force (N) was normalized to the calculated cross section area (CSA) of the diaphragm muscle bundles (*m* / l*d) where *m* is the muscle mass, *l* is the length*,* and *d* is mammalian skeletal muscle density (1.06 mg/mm) [26]. A fatigue protocol (30 Hz for 350 ms every 2 s for 5 min) was performed to determine the number of contractions required to reduce force to 60% of the force of the initial 30 Hz contraction.

### Diaphragm mitochondrial isolation

Diaphragm mitochondria were isolated following a standard method based on differential centrifugation. Briefly, diaphragm was immediately dissected upon collection and cut up finely with sharp scissors and diluted 1:10 (w/v) in ice-cold isolation medium consisting of 100 mM sucrose, 10 mM Tris base, KCl 50 mM, and 5 mM EDTA (pH 7.4). The minced tissues were homogenized with a Potter-Elvehjem homogenizer (three passages). The diaphragm homogenate was centrifuged 800 *g* for 10 min. The resulting supernatant was centrifuged at 1000 *g* for 10 min, filtered through cheesecloth, and centrifuged at 8700 *g* for 10 min to pellet mitochondria. Mitochondrial pellet was washed twice by suspension in isolation buffer and centrifuged at 8700 *g* for 10 min. Final pellet was suspended in 300 µL of isolation medium. The protein concentration of mitochondrial suspensions was determined using biuret method with bovine serum albumin as a standard.

### Mitochondrial oxygen consumption, ATP generation, and cytochrome c oxidase activity

Maximal oxygen consumption was measured in a glass cell of 1.5 ml volume fitted with a Clark oxygen electrode (Rank Brothers Ltd, France), and thermostated at 37 °C. Mitochondria (0.5 mg of protein/mL) were incubated in respiratory medium containing 120 mM KCl, 5 mM KH_2_PO_4_, 1 mM EGTA, 2 mM MgCl_2_, 0.3% of bovine serum albumin, and 3 mM HEPES, pH 7.4. Substrate concentrations were either 5 mM succinate in the presence of 5 μM rotenone or 2 mM ascorbate plus 500 μM N,N,N’N’-tetramethyl-p-phenylenediamine (TMPD) in the presence of 3 μM myxothiazol.

Oxygen consumption and ATP synthesis were performed at 37 °C in respiratory medium supplemented with glucose (20 mM), hexokinase (3 U/mL) as previously described [15, 19]. Respiratory substrates were succinate (5 mM) in the presence of rotenone. The mitochondrial ATP synthesis was initiated by the addition of 100 µM of ADP.

### Mitochondrial ROS production

The rate of mitochondrial production of reactive oxygen species (H_2_O_2_) was measured at 37 °C in the respiratory medium following the rate of appearance of resorufin from Amplex Red with a Kontron SFM25 fluorescence spectrophotometer (SFM-25, Kontron Instrument, Augsburg, Germany) at excitation and emission wavelengths of 560 and 584 nm, respectively, as described previously [15, 19]. Reaction conditions were 0.2 mg of mitochondrial protein per ml, 6 U/mL of horseradish peroxidase, 1 µM of Amplex Red. Mitochondrial ROS was measured both in the absence (state 4) and in the presence (state 3) of 100 µM ADP. The reaction was initiated by the addition of 5 mM succinate in the absence of rotenone, to quantify the mitochondrial ROS generation by complex 1 through a reverse electron flow and complex 3. When ROS production returned to basal after all ADP was used (state 4), 2 µM of rotenone was added to determine the maximum rate of H_2_O_2_ production of complex 3 of the respiratory chain. Calibration of H_2_O_2_ production was obtained by the addition of a known amount of H_2_O_2_.

### Measurement of lipid peroxidation

The amount of lipid peroxides was estimated in gastrocnemius muscle, heart, and liver using a spectrophotometric method, measuring malondialdehyde (MDA) as thiobarbituric acid-reactive substance (TBARS) following the method described by Ohkawa et al. [27]. Results are expressed as nmol MDA/mg tissue.

### Measurements of interleukin 1 beta (IL-1β) mRNA level in diaphragm

Total RNA was extracted from diaphragm muscle using the TriZol Reagent. Total RNA was further treated with DNase I (Invitrogen, Carlsbad, CA, USA) according to manufacturer recommendations. The integrity of the purified RNA was verified by agarose gel electrophoresis followed by ethidium bromide staining. Reverse transcription (RT) was performed using random hexamers as primers, reverse transcriptase (Superscript II), and 2 µg of total RNA in a total volume of 50 µl. Stability of expression levels under experimental and control conditions were investigated for the 18S ribosomal RNA (18S RNA) that served as a housekeeping gene. PCR was performed using standard protocols with SyBR Green PCR Supermix as a fluorescent detection dye in a real time iCycler (Bio-Rad, Hercules, CA, USA). We used a 2-step PCR amplification protocol with an annealing temperature of 60 °C for up to 40 cycles. Copy number was calculated and a standard curve was obtained with known amounts of target DNA. 18S mRNA was used as internal control for each sample. All PCR reactions for a given sample were performed in duplicates. The sequences of the PCR primers used were: IL-1β (gene bank reference NM_031512.2): 5′-GGC TGA CAG ACC CCA AAA GA-3′ (forward) and 5′-TTG TCG AGA TGC TGC TGT GA-3′ (reverse); 18S rRNA (gene bank reference NR_046237.1): 5′-TGA GGC CAT GAT TAA GAG GG-3′ (forward) and 5′-AGT CGG CAT CGT TTA TGG TC-3′ (reverse).

### Mitochondrial cytochrome c oxidase subunit 1 content

Equal amounts of diaphragm mitochondrial protein (100 μg estimated by bicinchoninic acid methods BCA Pierce France) were separated in 13% SDS-PAGE gels and transferred at a constant voltage to polyvinylidene fluoride (PVDF) membrane (Immobilon-P, Millipore) at 4 °C. After transfer, membranes were rinsed briefly in distilled water and incubated in Ponceau S solution (0.5% w/v in 1% v/v acetic acid) for 2 min followed by a brief rinse in distilled water, so that the lanes and bands were clearly visible. Then the protein transfer membranes were blocked in Tris buffered saline (TBS) with 0.1% Tween-20 and 5% non-fat dry milk overnight, and then membranes were incubated for 1 h at room temperature in TBS Tween containing the primary antibody against cytochrome c oxidase subunit 1 (1:1000 Invitrogen Cergy Pontoise France). Protein immunoreactivity was determined by chemiluminescence. Quantification of the signal intensity was determined on scanned films by using NIH image 1.63. Finally, the ratio of cytochrome c oxidase subunit 1 was expressed numerically as integrated optical density arbitrary units.

### Statistical analysis

All data are reported as means ± SEM. For the animal protocols, differences between groups were initially tested by non-parametric Kruskal–Wallis test with post hoc application of the Mann–Whitney test where appropriate. Statistical significance was defined as *p* < 0.05.

## Results

### Hemodynamics, gas exchanges, metabolic and proinflammatory cytokines parameters

**Fig. 1:**
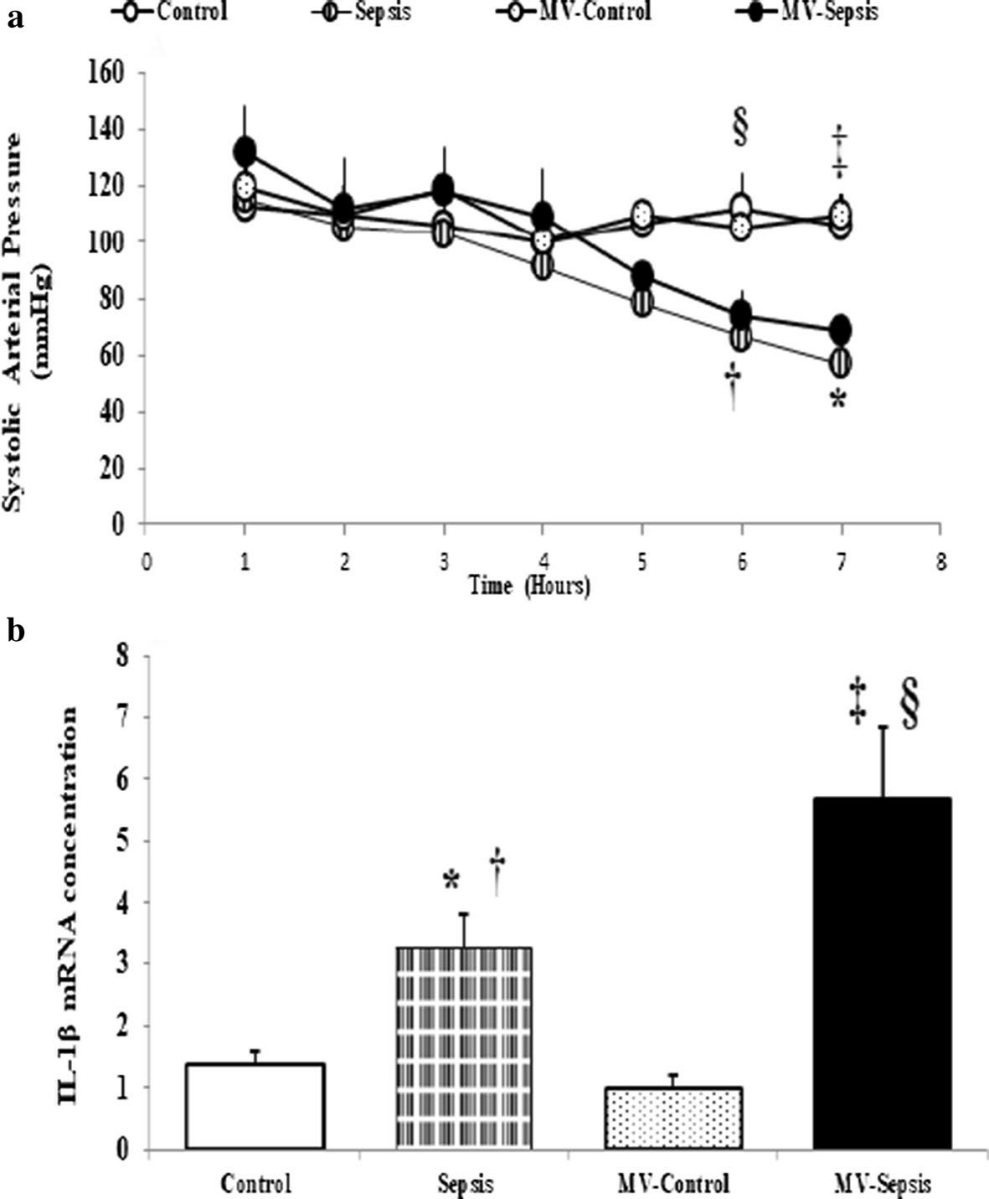
Effects of cecal ligation and puncture protocol and mechanical ventilation on arterial blood pressure, cytokines, and mRNA levels. Rats underwent cecal ligation and puncture (CLP) (*n* = 12) or sham operation (*n* = 12) at time 0. Animals were resuscitated with normal saline (10 ml.kg^−1^.h) intravenously. Half of the CLP and sham rats were intubated and mechanically ventilated and assigned to one of four groups (*n* = 6 per group): (1) a spontaneously breathing control group (control), (2) a spontaneously breathing septic group (sepsis), (3) a mechanically ventilated control group (MV-control), and (4) a mechanically ventilated septic group (MV-sepsis). **a** Shows the mean arterial blood pressure (MAP mmHg) throughout the study in control groups (open circles) and sepsis groups (dark circles). Data are means ± SEM from 6 animals of each group. *p < 0.05 sepsis versus control; ^‡^ < 0.05 MV-sepsis versus control; ^†^*p* < 0.05 sepsis versus MV-control; ^§^*p* < 0.05 MV-sepsis versus MV-control. Panel B shows diaphragm interleukin 1 beta (IL-1β) mRNA expression (**b**). Transcript levels of each group were standardized with mRNA level of 18 S. Data are means ± SEM from 4 animals per group. *p < 0.05 sepsis versus control; ^‡^ < 0.05 MV-sepsis versus control; ^†^*p* < 0.05 sepsis versus MV-control; ^§^*p* < 0.05 MV-sepsis versus MV-control for 4 animals per group

A marked hypotension occurred in the septic groups but not in the control groups. In the sepsis groups, hypotension appeared 240 ± 40 min after the CLP and intensified until the end of the recording period (Fig. 1a).

Rats from the sepsis groups also showed a profound metabolic acidosis along with an increased plasma lactic acid (Table 1) compared to controls. Finally, rats from sepsis groups exhibited higher IL-1β mRNA expression than controls (Fig. 1b), confirming that the CLP protocol induced a metabolic disorder associated with an upregulation of proinflammatory cytokines.

**Table 1:** Arterial blood gases (pH, PaO2, PaCO2, HCO3-) and plasma lactic acid measured at the end of the procedure in control, sepsis, MV-sepsis and MV-control groups

	Control	Sepsis	MV-control	MV-sepsis
pH	7.41±0.16	7.21±0.03*^†^	7.47±0.06	7.25±0.03^§‡^
PO_2_ (mmHg)	107 ± 15	108±18	146±3	145±9
PCO_2_ (mmHg)	35±2	17±3*†	41±7	23±3^§‡^
HCO_3_^−^ (mmol/L)	35±4	17±3*^†^	29±5	10±2^§‡^
Lactic acid (mmol/L)	2.99±0.95	9.32±1.86*^†^	2.11±0.46	9.85±1.44^§‡^

Data are means ± SEM from 6 animals per group. Data are means ± SEM from 6 animals per group. *p < 0.05 sepsis versus control; ^†^*p* < 0.05 sepsis versus MV-control; ^‡^*p* < 0.05 MV-sepsis versus control and ^§^*p* < 0.05 MV-sepsis versus MV-control

### Isometric force and fatigue

**Fig. 2:**
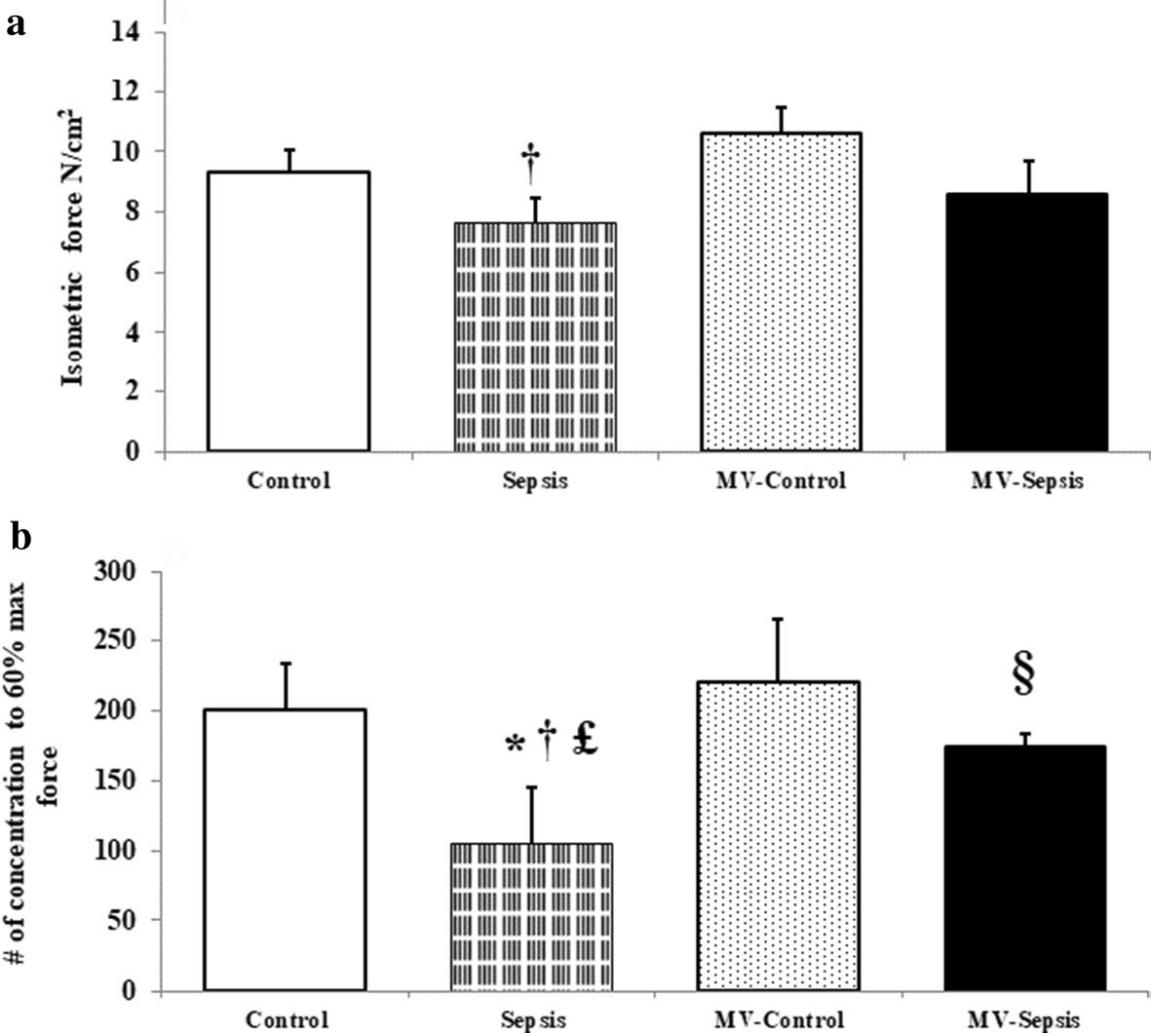
Diaphragm isometric force and diaphragm fatigue. Isometric force at 70 Hz stimulation taken from force–frequency analysis in diaphragm strips from sepsis, control, MV-control and MV-sepsis (**a**). Data are means ± SEM from 4 animals per group. ^†^*p* < 0.05 sepsis versus MV-control; Number of contraction to reduce force to 60% of initial isometric force when isolated diaphragm strips underwent a fatiguing stimulation protocol (30 Hz for 350 ms every 2 s for 5 min) (**b**). Data are means ± SEM from 4 animals per group. *p < 0.05 sepsis versus control; ^†^*p* < 0.05 sepsis versus MV-control; ^£^*p* < 0.05 MV-sepsis versus sepsis, for 4 animals per group

Isometric force generated by the diaphragm strips was lower in the CLP groups (Fig. 2a), but it reached significance only when compared the spontaneously breathing sepsis rats group with the MV-control group (*p* = 0.0372). CLP significantly increased diaphragm fatigue in spontaneously breathing rats. In response to a fatiguing stimulation protocol, we found that spontaneously breathing sepsis group diaphragms required on average 104 ± 8 contractions to reach 60% maximum isometric force at 30 Hz compared to 175 ± 8 (*p* = 0.038) in the MV-sepsis group, 200 ± 34 (*p* = 0.026) in spontaneously breathing control group, and 220 ± 45 (*p* = 0.028) in the MV-control group (Fig. 2b).

### Effect of CLP procedure and mechanical ventilation on mitochondrial respiration and ATP production

In the spontaneously breathing animals, CLP decreased mitochondrial ADP stimulated respiration (–40%) and ATP production (–55%), compared to controls (Table 2). The effects of CLP on mitochondrial oxygen consumption and ATP production were reversed by mechanical ventilation: mitochondrial oxygen consumption was 26% higher (*p* = 0.021) and ATP production was (44%) higher (*p* = 0.039) in the MV-sepsis group compared to the spontaneously breathing sepsis group (Table 2). However, mitochondrial efficiency as shown by ATP/O and RCR was not affected by any of the treatment (Table 2). The CLP protocol had no effect on the mitochondrial state 4 respiration (Table 2). Finally, the mechanical ventilation had no effect on the mitochondrial oxygen consumption and ATP production of control animals.

**Table 2:** Mitochondrial oxygen consumption and ATP synthesis

	Control	Sepsis	MV-control	MV-sepsis
State 3 respiration (natm/min/mg)	42±3	25±3*^†₤^	40±2	34±4
State 4 respiration (natm/min/mg)	12.8±3.9	6.2±1.1	11.1±1.5	8.8±2.4
RCR	4.18±0.81	4.32±0.53	3.83±0.55	4.04±0.72
ATP production	119±21	53±15*^†₤^	130±23	95±10
ATP/O	2.75±0.50	2.20±0.60	3.23±0.62	2.86±0.41

Measures were performed on diaphragm mitochondria from rats of the four following groups: spontaneously breathing controls (Control), spontaneously breathing sepsis group (Sepsis), mechanically ventilated control group (MV-control) and a mechanically ventilated sepsis group (MV-sepsis). Mitochondria were energized with succinate as respiratory substrate in the presence of rotenone State 3 phosphorylating respiration as well as ATP production were measured at 100 µM of exogenous ADP. State 4 respiration was obtained by inhibiting the ATP synthase with 3 µg/ml of oligomycin. Data are means ± SEM from 6 animals per group. *p < 0.05 sepsis versus Control; ^†^*p* < 0.05 sepsis versus MV-control; ^£^*p* < 0.05 sepsis versus MV-sepsis

### Effect of CLP procedure and mechanical ventilation on cytochrome c activity and content

**Fig. 3:**
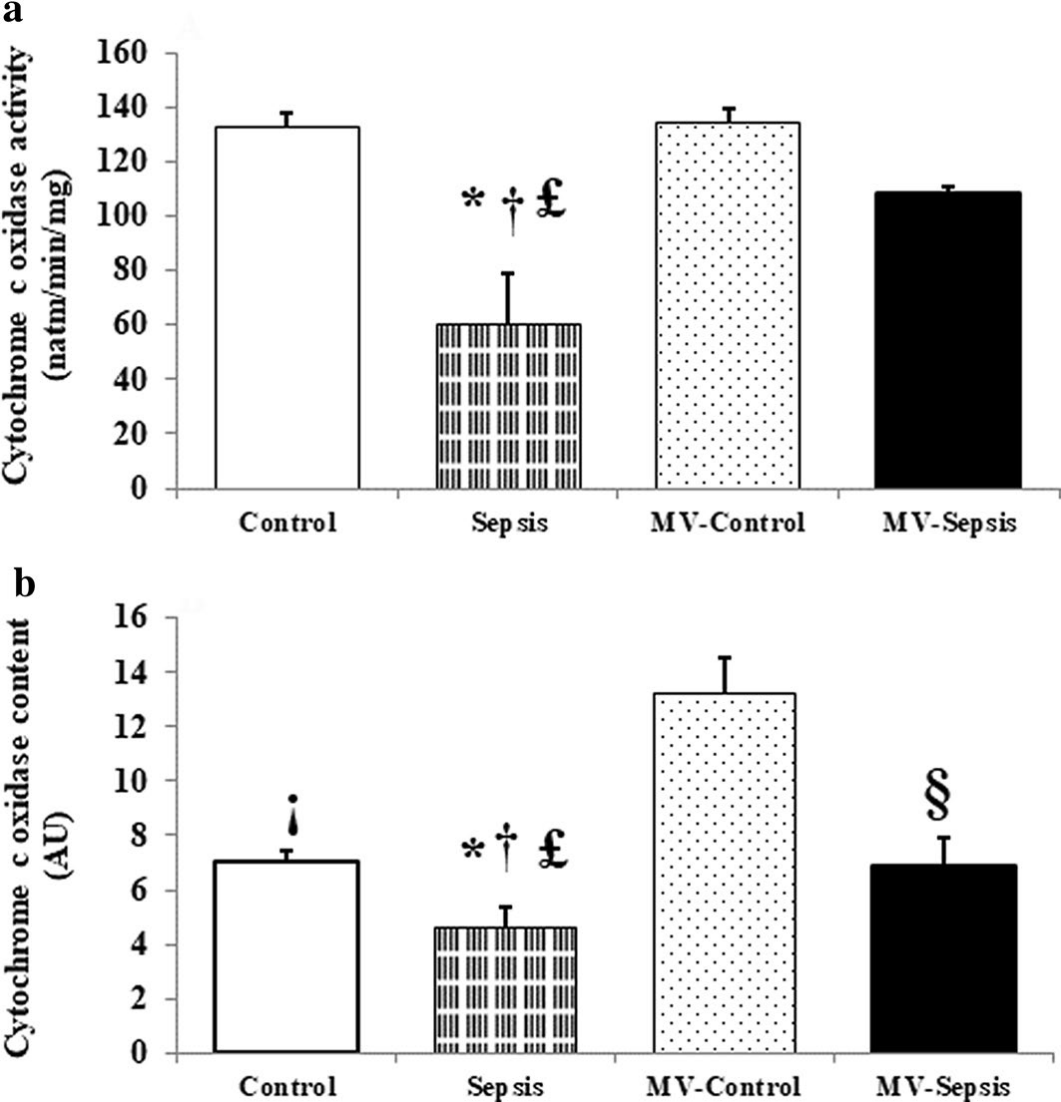
Cytochrome c oxidase activity and subunit 1 content. The maximal activity of cytochrome c oxidase (**a**) and representative western blot of cytochrome c oxidase subunit 1 (**b**). Cytochrome c oxidase was assessed by the addition of 2 mM ascorbate plus 500 μM N,N,N’N’-tetramethyl-phenylenediamine (TMPD) in the presence of 3 μM myxothiazol. Values are means ± SEM from 6 animals in control, sepsis, MV-sepsis, and MV-control. *p < 0.05 sepsis versus control; ^†^*p* < 0.05 sepsis versus MV-control; ^£^*p* < 0.05 sepsis versus MV-sepsis. Western blot analysis of diaphragm mitochondrial cytochrome c oxidase subunit 1 (Panel B). Data are means ± SEM from 4 animals per group. *p < 0.05 sepsis versus control; ^†^*p* < 0.05 sepsis versus MV-control; ^£^*p* < 0.05 sepsis versus MV-sepsis; ^¡^*p* < 0.05 control versus MV-control; ^§^*p* < 0.05 MV-sepsis versus MV-control

The CLP procedure was associated with decreased mitochondrial cytochrome c oxidase activity in the spontaneously breathing group compared to controls (–54% *p* = 0.027) (Fig. 3a). Such inhibition of cytochrome c oxidase activity in the spontaneously breathing sepsis group mirrored a downregulation of cytochrome c oxidase subunit 1 content (–69% *p* = 0.039) compared to controls.

MV of CLP rats restored the levels of cytochrome c oxidase activity and content back to that of controls. Accordingly, the activity of mitochondrial cytochrome c oxidase was higher in the MV-sepsis group compared to the spontaneously breathing sepsis group (+ 45%, *p* = 0.045) (Fig. 3a). The level of cytochrome c oxidase subunit 1 content was higher in the MV-sepsis group compared to the spontaneously breathing sepsis group (+ 72% *p* = 0.045) (Fig. 3b).

### Effect of CLP and mechanical ventilation on mitochondrial H_2_O_2_ production and diaphragm MDA

**Fig. 4:**
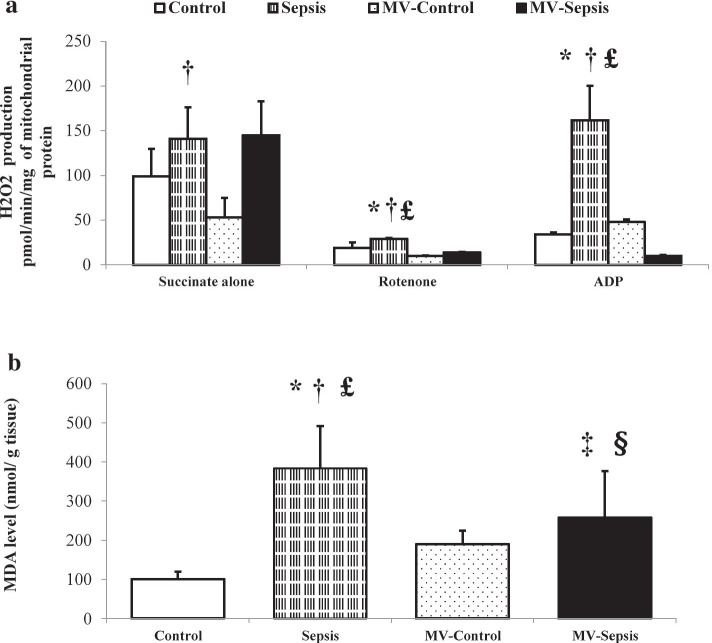
Diaphragm mitochondrial H_2_O_2_ production and MDA content. **a** Shows H_2_O_2_ measured in the presence of 0.3% free fatty acid–BSA and 5 mM succinate without rotenone (succinate), then ADP (succinate-state 3) added to increase mitochondrial activity ATP generation and inhibit H_2_O_2_ generation, and then rotenone (rotenone) added to measure radical production at the level of complex 3, as described in “Materials and methods” section. Data are means ± SEM from 4 animals per group. *p < 0.05 sepsis versus control; ^†^*p* < 0.05 sepsis versus MV-control; ^£^*p* < 0.05 sepsis versus MV-sepsis. **b** Shows MDA (malondialdehyde) content in the diaphragm of control, sepsis, MV-sepsis, and MV-control. Six hours after CLP, diaphragms were removed to determine thiobarbituric acid-reactive species content. Data are means ± SEM from 6 animals per group. *p < 0.05 sepsis versus control; ^†^*p* < 0.05 sepsis versus MV-control; ^‡^*p* < 0.05 MV-sepsis versus control; ^§^*p* < 0.05 MV-sepsis versus MV-control; ^£^*p* < 0.05 sepsis versus MV-sepsis.

Mitochondrial H_2_O_2_, generated by complex 1 through reverse electron flow and complex 3 by forward electron flow, was observed by incubating mitochondria with succinate in the absence of rotenone. The maximum rates of H_2_O_2_ release measured under basal non-phosphorylating respiration (succinate alone) were higher in CLP groups compared to control groups (Fig. 4a). This higher rate reached significance when we compared the spontaneously sepsis group with the MV-control group (*p* = 0.00149). Mitochondrial H_2_O_2_ production was mostly abolished by the addition of ADP (active respiration state) in the MV-sepsis group (*p* = 0.034) and control group (*p* = 0.089) and MV-control group (*p* = 0.0152) compared to the spontaneously breathing sepsis group. Interestingly, when the H_2_O_2_ released from complex III was measured in the presence of rotenone, we found a higher value of H_2_O_2_ generated by the mitochondria of spontaneously breathing sepsis group than the other groups, showing that MV can lower mitochondrial H_2_O_2_ generation from CLP rats at the level of complex 3.

The diaphragm MDA contents showed a similar pattern with mitochondrial H_2_O_2_ production at the level of mitochondrial complex 3. The diaphragm MDA contents were higher in the spontaneously breathing sepsis group compared to the MV-sepsis group (*p* = 0.028), spontaneously breathing control (*p* = 0.027), and MV-control group (*p* = 0.021) (Fig. 4b).

## Discussion

We reported that mitochondrial respiration and ATP production were decreased in the diaphragm of septic rats, but partially restored by MV. This protective effect was associated with preserved mitochondrial cytochrome c oxidase activity and content and ATP generation. Moreover, MV prevented the increase in mitochondrial oxygen radical production at complex 3 level and the oxidative damage to lipid accumulation observed in the diaphragms of the spontaneously breathing sepsis group. However, MV did not improve contractile properties of the diaphragm muscle during sepsis.

In this study, we used a hypotensive CLP model of severe sepsis that is widely used to investigate the physiologic derangement at the early phase of septic disease in spontaneously breathing [14, 19, 21] or mechanically ventilated animals [20, 28, 29]. One may question the relevance of studying diaphragmatic function from a short-term sepsis and MV experimental model. Two studies [9, 30] have shown that proteolysis, oxidation and autophagy markers are present on the 6th hour of MV. If we consider that those markers predict muscle atrophy, then our biochemical results must be considered. Moreover, a short-term CLP model of sepsis upregulated IL-1β mRNA [15, 19, 21, 31]. In accordance with previous studies using the same severe model of sepsis [19–21, 28, 29], we observed an early arterial hypotension within the first 3 h (Fig. 1a). Such hypotension following CLP-induced peritonitis was accompanied by upregulated expression of proinflammatory cytokines in the diaphragm, which may link the diaphragm dysfunction to the peritonitis. Among the early upregulated proinflammatory cytokines [31], IL-1β mRNA persist after the 4th hour of CLP [21, 31] and has been shown to overwhelm inflammatory response and potentiated mitochondrial dysfunction and oxidative stress through the inflammasome [32, 33]. Accordingly, we observed a decrease in muscle maximal force which mirrored the increase in IL-1β mRNA gene expression in the spontaneously breathing sepsis group (Figs. 2a and 1b). Note that the low cytokine concentration in the MV-control group excluded the participation of MV in the proinflammatory reaction occurring in the diaphragm of the present short-term sepsis model. Although we cannot completely rule out the possibility that low blood flow may contribute to diaphragm dysfunction, there are several pathways by which the upregulation of IL-1β mRNA could be associated with the decrease in diaphragm force and increase in fatigue. These include activation of proteolysis [34] and/or IL-1β mRNA in the diaphragm muscle and its potential toxic effects on muscle fibers [35].

Since the founding work of Hussain et al. [2] showed that endotoxin infusion leads to respiratory failure and death, several lines of evidence have shown a relationship between diaphragm weakness following proinflammatory activation of nitric oxide synthase (iNOS) and mitochondrial dysfunction [36, 37]. Even though IL-1β may induce diaphragm weakness without any oxidative stress [35], our data along with previous findings [15, 19, 21, 38] suggest that the intra-diaphragmatic upregulation of IL-1β mRNA leads to an increase in oxidative and nitrosative stress, possibly linked to the stimulation of iNOS and inhibition of mitochondrial respiration and ATP generation [36, 37]. With regard to those studies, we suggested that the early IL-1β mRNA activation would trigger diaphragm dysfunction through its negative effect upon mitochondrial function and ROS generation [4]. We confirmed here that CLP impaired diaphragm mitochondrial respiration and ATP production in the early stages of septic disease. In particular, we observed a decrease in oxygen consumption driven by FADH-mediated respiration (complex 2, Table 2) and ascorbate-TMPD-driven respiration (cytochrome c oxidase activity-complex 4) in the spontaneously breathing septic diaphragm (Fig. 3). This decreased oxidative capacity was associated with decreased ATP production and increased H_2_O_2_ production, but did not significantly alter mitochondrial efficiency (RCR and ATP/O).

The early alteration of mitochondrial function in the spontaneously breathing sepsis group may be mechanistically explained by the inhibition and depletion of cytochrome c oxidase (complex 4). Published evidence has shown that cytochrome c oxidase is the first target of the mitochondrial respiratory chain during sepsis [15, 19, 38, 39]. The effects of sepsis on cytochrome c oxidase are particularly pronounced when electrons are provided to the mitochondrial electron transport chain by the complex 2 rather than complex 1 substrates [19, 40]. If subunit 1 of mitochondrial cytochrome c oxidase were inhibited by proinflammatory cytokines and nitric oxide [38, 39], it would be unable to lower mitochondrial H_2_O_2_ generation at mitochondrial complex 3 [41]. The resulting increase in H_2_O_2_ generation and activation of intra-diaphragmatic iNOS may then explain mitochondrial protein and lipids nitration/oxidation and depletion [36, 40, 42].

Oxidation and nitration of mitochondrial proteins in the diaphragm may be a consequence of oxidative stress that causes mitochondrial dysfunction and diaphragm weakness [12, 36, 40]. The decrease in cytochrome c oxidase activity associated with the marked depletion of its subunit 1 content (Fig. 3), together with the increased in mitochondrial H_2_O_2_ generation and MDA accumulation in the diaphragm (Fig. 3), aligns with those studies. The mechanism could be the concomitant increase in mitochondrial superoxide generation and nitric oxide generation, leading to peroxynitrite responsible for irreversible mitochondrial inhibition [36]. Therefore, during sepsis, mitochondria of the diaphragm seem to be the main target of their own ROS generation with deleterious effects on their contractile properties [4]. Interestingly, the early infusion of antioxidants has been shown to lower the free radical generation and MDA contents and improve diaphragm function in CLP rats [12, 14].

The most innovative result of our study is that MV in the CLP group restored diaphragm mitochondrial oxygen and ATP fluxes to that of the control group. MV also lowered mitochondrial ROS generation and prevented oxidative stress by reducing MDA content in the sepsis group. This effect occurred through the preservation of cytochrome c oxidase function and content, confirming the role of cytochrome c oxidase in anti-oxidant defense [15, 18]. As shown in Table 2, the mitochondrial oxygen consumption was higher in the MV-sepsis group compared to the spontaneously breathing sepsis group. Higher oxygen consumption in the MV-sepsis group compared to the spontaneously breathing sepsis group was also associated with higher ATP generation. However, MV had no significant effect on state 4 respiration, suggesting that changes in mitochondrial properties during sepsis and MV were not driven by alterations of mitochondrial membrane proton leak [15]. However, if MV maintained a high rate of mitochondrial ATP generation, it did not prevent the diaphragm strength from being altered during sepsis. Thus, the low contractile activity of the diaphragm in the MV-sepsis rats group could result from muscle proteolysis associated with sepsis and/or MV rather than by a lack of ATP generated by the mitochondria which is in line with the work of Marloes Van den Berg, et al. [11]. Our study showed that MV affected the diaphragm mitochondria during sepsis in two ways: (i) MV kept mitochondrial oxidative and phosphorylative capacities intact, and (ii) MV decreased ROS generation in spite of increased in mitochondria activity. This double effect occurred through the preservation of mitochondrial cytochrome c oxidase function and content, which increased ATP production [16] and decreased ROS generation [18] under stress conditions. This beneficial effect of MV on cytochrome c oxidase content and activity could be explained by HIF 1 α stabilization induced by MV [43]. This has been ascribed to diaphragm hypoperfusion at the early stage of MV [43] and has shown to decrease ROS generation and improved mitochondrial function [41]. Cytochrome c oxidase is the terminal oxidase of the mitochondrial electron transport chain that catalyzes the oxidation of cytochrome c and the reduction of dioxygen to water. Cytochrome c oxidase consists of 13 subunits and regulates mitochondrial respiration and efficiency [15–17, 19]], proton translocation, and ROS generation [18]. Changes in enzymes stoichiometry can be related to protein depletion, catalytic site inhibition, or downregulation of RNA synthesis protein [24, 38, 39, 42] and may therefore decrease the oxygen consumption at the cytochrome c oxidase level and increase the mitochondrial ROS generation [15, 18, 19]. The depletion of cytochrome c oxidase subunit 1 found in the diaphragm of the spontaneously breathing sepsis group (Fig. 3) and in the liver mitochondria during sepsis [15, 19] highlights the crucial role of cytochrome c oxidase on mitochondrial respiration and ROS generation. Indeed, the increase in ROS generation by the mitochondria from spontaneously breathing sepsis group could be explained by the slowing of the electron flow at cytochrome c oxidase level which sub unit 1 is depleted in the previous [15, 19] and the present study. This slowdown results in a direct reaction between electrons from mitochondria complexes 1, 2 and especially 3 with the molecular oxygen. Preservation of cytochrome c oxidase content induced by MV improved its efficiency and explained the low ROS generated at complex 3 level and the consecutive decrease in the oxidative damage (MDA) (Fig. 3). A similar trend has been described for heart function after septic rats have received cytochrome c treatment [44]. Therefore, cytochrome c oxidase is the first target of septic insult [15, 19, 36] and mediator of mitochondrial dysfunction and oxidative damage to tissue due to its lack of ROS regulation [15, 18].

Our study also showed that MV decreased radical generation was associated with decreased oxidative damage to lipids in the diaphragm contrary to Li et al. study [9]: the MDA content was lower in the MV-sepsis group compared to the spontaneously breathing sepsis group (Fig. 4b). With regard to the main role of oxidative stress in diaphragm weakness during sepsis, the curative benefits of the early institution of MV on the septic diaphragm proposed by Laghi [6] could be found in the present work at the mitochondria level. Indeed, we showed here that, in addition to its ability to lower free radical generation and oxidative damage as a free radical scavenger, MV restored mitochondria capabilities to generate ATP.

Another non-exclusive explanation for our results regarding the effects of MV may be linked to muscle paralysis by the drug atracurium besilate. Atracurium is a skeletal muscle relaxant and has been shown to decrease muscle and serum inflammatory response and to improve diaphragm function in experimental sepsis [45]. We used this medication in our experiment to avoid excessive muscle activity [22]. However, contrary to what was previously reported, we found no significant improvement in diaphragm force or diaphragm inflammatory response (Figs. 2a and 1b). We believe that the paralysis may have decreased the overall muscle ATP demand and thereby reduced mitochondrial activity, and that this could have decreased ROS generation and oxidative damage in the diaphragm and preserved the activity of the cytochrome c oxidase. However, we did not find a difference between the control groups regarding mitochondrial function and ROS generation.

## Conclusion

The early institution of MV during experimental severe sepsis partially restores mitochondrial function, and does not exacerbate diaphragm weakness.

## Data Availability

The datasets used and/or analyzed during the current study are available from the corresponding author on reasonable request.

## Abbreviations

CLP: Cecal ligation and puncture
ALT: Alanine amino transferase
CLP: Cecal ligation and puncture
CcO1: Cytochrome c oxidase subunit 1
FCCP: Carbonyl cyanide-p-tri-fluoro-methoxy-phenyl-hydrazone
IQR: Interquartile range
TMPD: N,N,N’,N’-Tetramethyl-1,4-benzenediamine dichloride
TPMP: Methyl-tri-phenyl-phosphonium
ATP: Adenosine triphosphate
ROS: Reactive oxygen species
